# Three Pathological Cases

**Published:** 1897-07

**Authors:** I. P. Wilson


ARTICLE XI.

### THREE PATHOLOGICAL CASES.

BY I. P. WILSON, D. D. S.

**Read before the Anniversary Clinic, Chicago Dental Society.**

Case I. Mrs. S——, aged twenty-eight, lived on a farm; had always enjoyed good health. Last April she suffered severely from nervous toothache in first left upper molar. Home remedies were applied, and after a time the pain subsided. The pulp had perished. Soon after the tooth became sore, the adjacent parts inflamed and the face swollen. After intense suffering for some days the acute form subsided, leaving a benumbed, heavy pain in the malar region.

A dentist in a neighboring town was then consulted, and the tooth extracted. This was followed by a gush of pus from the antrum of a most sickening character, causing nausea during the rest of the day. The discharge continued through the alveolus for nearly a week, when there was a cessation of pain, and finally the socket closed. But little discharge had at any time taken place through the nose. Some months passed, when the pain in the cheek and in the ethmoid region again appeared. She grew rapidly worse, and after receiving medical treatment for some time with no beneficial results, she returned to her parents' home in our city, that she might have her mother's care.

Other physicians were summoned, but were unable to find the hidden cause of the disease, or make a satisfactory diagnosis. In two weeks from the time of her removal she died of septicemia.

A post-mortem examination was held, the brain removed and the horizontal plate of the ethmoid broken down when the cells of that bone were found to be infiltrated with corrupt matter. The two large air cavities of the sphenoid were also filled with pus. The frontal sinuses were apparently in a normal condition. The examination was about to be concluded when one of the physicians kindly suggested that the case would be of peculiar interest to the writer, and asked that I be sent for.

On my arrival I inquired about the previous condition of her teeth, and was told that the dental organs had not been suspected as having anything to do with the disease. The antrum was then opened and found to be filled with a mass of thick, cheesy, corruption.

At the suggestion of the physician in charge, I accompamied him to another room to interview the mother, and from her we procured the foregoing history of the case. It is due the physicians who attended the lady in her last illness, to state that the apparent good health of the patient for a time after her first illness had led her family to dis-associate the first stage of the disease from her last sickness, and so her physicians were not advised of previous dental irritation.

I ought to state that the septum of the nose was deflected to such an extent as to almost entirely close the nostril on the affected side, so that the ostium from the antrum was almost if not entirely obstructed. This, of course, accounts largely for there being no discharge from the nose, and the consequent engorgement of the antrum.

Case. 2. Dr. D., aged thirty-seven, a clergyman, was advised by his physician to give up his work and travel, because of cerebral difficulty, softening of the brain being suspected. A trip abroad was planned, and in company with an attendant he sailed for London. Immediately on arrival, he was placed under the treatment of a noted physician of that city. A few weeks elapsed, when he complained of an aching tooth in the lower jaw. In a few days his face and neck became greatly swollen. His suffering was intense, and was attended with chills and fever.

A large poultice, covering the side of his face and neck, was ordered by his physician. Days passed without relief. A lancet was at length used, and a quantity of pus discharged. The swelling in the cervical region, however, only partially subsided. His condition grew rapidly worse, till death from septicemia relieved the sufferer.

His attendant was a man of intelligence, and of close observation, and from him I learned the above facts at the time of the burial at Bloomington in this State. I am satisfied that pus had pocketed at different places between the muscles of the submaxillary region. A dentist was not called, nor did the tooth receive any direct treatment.

In this case, as in the preceding one, there is much to be read "between the lines."

Case 3. Mrs. P., aged sixty-eight, of previous good health, and with excellent family history.

I was called in consultation with attending physicians. The case had been under treatment for more than a year. Morbid growths had been repeatedly removed, of late, from the nose. The cheek was considerably enlarged. A gold plate had been worn for two years, the rim of which was buried in the aggravated buccal tissues, making a cut nearly two inches in length, and a quarter of an inch in depth.

These symptoms led me to believe the antrum to be the seat of the disease.

At the request of the physicians, I opened into the cavity through the cut made in the soft tissues by the plate.

The posterior and superior portions of the sinus were found to be well filled with a spongy growth that bled freely on the slightest touch. There was no difficulty in irrigating the unoccupied portion of the cavity, the water passing freely into and out of the nose.

The plate was now, for the first time, laid aside.

Believing the disease to be of a malignant character, I advised the lady to go at once to a Chicago hospital for treatment at the hands of a skilful surgeon. But she and her friends, as well as the physician who had attended her, suggested that we wait for further development. I accordingly visited her home daily for three weeks, each time cleansing the passage from the antrum to the nose with medicated washes, which afforded considerable comfort at the time. I discovered, however, that the disease was rapidly gaining ground. Polypoid growths were continually forming in the nose, which her physician from time to time removed.

An angry appearing tumor of the cauliflower form, commenced bulging through the opening into the mouth. The cervical glands became involved.

After carefully studying the case in the light of our best authorities, I pronounced the disease to be of a carcinomatous nature, and to verify my conclusions I sent a clipping from the growth to a Chicago microscopist for examination, requesting a prompt reply, which came by wire in the following words: "Carcinoma—Prognosis grave."

I advised the friends of the unfortunate woman to take her at once to Chicago, which they did.

Prof. Senn was consulted, and confirmed the diagnosis given. An operation was determined on as the only possible hope of lengthening her life. An enlarged cervical gland was first removed, followed by excision of the left superior maxillary.

The patient rallied from the operation and appeared to be doing well for a few days, when facial erysipelas set in and death soon followed. Prof. Senn, I am informed, regarded the case of traumatic origin. Her brother, Dr. Smith, a prominent physician of Wellington, Ohio, who attended the operation, informed me by letter, received since writing the above, that his sister "had no heredity inviting the conditions and cause of her death."

The question of peculiar interest to us as dentists, is the cause of the malady. Was it the result of long and continued irritation, resulting from an ill-fitting plate? I am inclined to that opinion.—*Dental Review.*

